# Tuberculosis infection and disease in South African adolescents with perinatally acquired HIV on antiretroviral therapy: a cohort study

**DOI:** 10.1002/jia2.25671

**Published:** 2021-03-14

**Authors:** Lisa J Frigati, Katalin A Wilkinson, Stanzi le Roux, Karryn Brown, Sheena Ruzive, Leah Githinji, Wonita Petersen, Sabine Belard, Mark F Cotton, Landon Myer, Heather J Zar

**Affiliations:** ^1^ Department of Paediatrics and Child Health University of Cape Town Cape South Africa; ^2^ Family Center for Research with Ubuntu (FAMCRU) Department of Paediatrics and Child Health Stellenbosch University Cape Town South Africa; ^3^ Wellcome Centre for Infectious Disease Research in Africa Institute of Infectious Disease and Molecular Medicine University of Cape Town Cape Town South Africa; ^4^ The Francis Crick Institute London United Kingdom; ^5^ Division of Epidemiology and Biostatistics School of Public Health and Family Medicine University of Cape Town Cape Town South Africa; ^6^ Department of Pediatric Pulmonology, Immunology and Intensive Care Medicine Charité ‐ Universitätsmedizin Berlin Berlin Germany; ^7^ Berlin Institute of Health Berlin Germany; ^8^ SAMRC Unit on Child and Adolescent Health University of Cape Town Cape Town South Africa

**Keywords:** tuberculosis, coinfection, incidence, adolescents, perinatal, HIV

## Abstract

**Introduction:**

There are limited data on Tuberculosis (TB) in adolescents with perinatally acquired HIV (APHIV). We examined the incidence and determinants of TB infection and disease in the Cape Town Adolescent Antiretroviral Cohort (CTAAC).

**Methods:**

Youth between nine and fourteen years on antiretroviral therapy (ART) for more than six months in public sector care, and age‐matched HIV‐negative adolescents, were enrolled between July 2013 through March 2015 and followed six‐monthly. Data were censored on 31 October 2018. Symptom screening, chest radiograph, viral load, CD4 count, QuantiFERON (QFT) and sputum for Xpert MTB/RIF, microscopy, culture and sensitivity were performed annually. TB infection was defined by a QFT of >0.35 IU/mL. TB diagnosis was defined as confirmed (culture or Xpert MTB/RIF positive) or unconfirmed (clinical diagnosis and started on TB treatment). Analyses examined the incidence and determinants of TB infection and disease.

**Results:**

Overall 496 HIV+ and 103 HIV‐negative participants (median age at enrolment 12 years (interquartile range, IQR 10.6 to 13.3) were followed for a median of 3.1 years (IQR 3.0 to 3.4); 50% (298/599) were male. APHIV initiated ART at median age 4.4 years (IQR 2.1 to 7.6). At enrolment, 376/496 (76%) had HIV viral load <40 copies/mL, median CD4 count was 713 cells/mm^3^ and 179/559 (32%) were QFT+, with no difference by HIV status (APHIV 154/468, 33%; HIV negative 25/91, 27%; *p* = 0.31). The cumulative QFT+ prevalence was similar (APHIV 225/492, 46%; 95%CI 41% to 50%; HIV negative 44/98, 45%; 95% CI 35% to 55%; *p* = 0.88). APHIV had a higher incidence of all TB disease than HIV‐negative adolescents (2.2/100PY, 95% CI 1.6 to 3.1 vs. 0.3/100PY, 95% CI 0.04 to 2.2; IRR 7.36, 95% CI 1.01 to 53.55). The rate of bacteriologically confirmed TB in APHIV was 1.3/100 PY compared to 0.3/100PY for HIV‐negative adolescents, suggesting a fourfold increased risk of developing TB disease in APHIV despite access to ART. In addition, a positive QFT at enrolment was not predictive of TB in this population.

**Conclusions:**

High incidence rates of TB disease occur in APHIV despite similar QFT conversion rates to HIV‐negative adolescents. Strategies to prevent TB in this vulnerable group must be strengthened.

## 1 Introduction

Adolescence is a period of increased risk for both *Mycobacterium tuberculosis (Mtb)* infection and tuberculosis disease (TB), compared to the pre‐adolescent period [1, 2]. Globally, an estimated 1.8 million people between the ages of 10 to 24 years developed TB in 2012, with 534 000 of these living in Africa [3, 4]. Adolescents are also more likely to be infectious (smear positive) than younger children [5]. HIV is a risk factor for *Mtb* infection and TB disease. Countries with a high HIV prevalence have a substantive TB disease burden including in adolescents [6]. Although access to antiretroviral therapy (ART) has led to a decrease in TB incidence in adolescents and children with HIV, they remain at increased risk of TB compared to those who are HIV negative [7, 8, 9, 10]. Indeed, TB remains a major cause of hospitalization and mortality in adolescents living with HIV [11]. Furthermore, TB may have long‐term consequences on lung health with subsequent impairment of lung function [12].

*Mtb* infection can be measured using Interferon Gamma Release assays (IGRA), for example Quanti‐FERON (QFT), a positive test denotes TB infection, however, further signs and symptoms and bacteriological evidence needs to be sought before the diagnosis of “TB disease” is made. Previous estimates of *Mtb* infection prevalence among adolescents in sub‐Saharan Africa have indicated high annual risks among the general population but often lack data on HIV status [13, 14]. In addition, a recent review that focused on bacteriologically confirmed pulmonary Tuberculosis (PTB) in adolescents and youth found no studies among those with HIV [15]. This may be due to challenges in confirming TB in high burden settings. Adolescents with perinatally acquired HIV (APHIV) are likely to be a specifically vulnerable group given risks of disengagement from care and ART treatment fatigue [16]. There is therefore a clear and urgent need to understand the burden of HIV‐associated TB in adolescents in high HIV and TB prevalence settings, utilizing robust data collection for TB diagnosis and measures of HIV severity. To address this critical knowledge gap, we investigated the incidence and clinical factors associated with TB infection and disease in a South African cohort of APHIV on ART compared to a matched HIV‐negative group.

## 2 Methods

### 2.1 Study population and design

This analysis draws on data from the Cape Town Adolescent Antiretroviral Cohort (CTAAC), a prospective study that enrolled APHIV between 01 July 2013 and 31 March 2015. Children and adolescents aged nine to fourteen years already accessing HIV care at one of seven public service sites in the Western Cape Province, South Africa were eligible for enrolment, provided they had been on ART for at least six months and were aware of their HIV status. Concurrently, a comparison group of HIV negative (HIV−) adolescents from primary care facilities in the same communities were enrolled, frequency‐matched on sex and age. We excluded HIV− youth with chronic/ systemic inflammatory conditions or known chronic neurological, pulmonary or cardiovascular disease. HIV− status was confirmed at enrolment, with annual retesting thereafter, following informed consent.

Sociodemographic data were collected at enrolment and the participant’s clinical record was reviewed. Thereafter, participants were seen biannually at the study site. A structured questionnaire and physical examination including Tanner staging, World Health Organization (WHO) HIV staging and anthropometry were performed at enrolment and annually. Body Mass Index (BMI) was calculated as weight in kilograms divided by height in metres squared (kg/m^2^) and classified by WHO reference standards [17]. At each study visit, screening for signs and symptoms of TB was performed; if symptomatic, participants had a chest radiograph (CXR). In addition, two sputum specimens (induced /expectorated) were collected at enrolment, annually and if an intercurrent pneumonia was suspected, and sent for Xpert MTB/RIF (Cepheid) and microscopy, liquid culture using semi‐automated 7H9 broth‐based Mycobacterial Growth Indicator Tubes (MGIT960; Becton Dickinson, Sparks, MD). Any participant diagnosed with TB at a study visit was referred for treatment at their site of routine clinical care.

At enrolment, all participants received Quanti‐FERONTB Gold In‐Tube^®^ (QFT; Qiagen, Hilden, Germany) testing. For participants with a positive QFT at enrolment or subsequently, the test was not repeated. Provided the prior QFT tests were negative or indeterminate, QFT was repeated annually for three years. QFT tests were not done in real time, but were stored and batched tested, so results were not able to inform participant care. Additional laboratory measures performed at enrolment and annually included HIV viral load (Roche COBAS Ampliprep/Taqman) and CD4 cell count (Beckman Coulter ^®^, Brea, CA, USA) in HIV+ participants; HIV− participants were retested every six months for HIV infection, using Alere Determine ^TM^ (Abbott, Chiba, Japan).

Throughout study follow‐up, participants continued to receive routine care at their primary care sites, including ART and prophylaxis against opportunistic infections (OI). The most commonly used ART regimens, according to national guidelines were Abacavir, Lamivudine and either Efavirenz or a protease inhibitor. According to national guidelines, isoniazid (INH) prophylaxis is given to children living with HIV between the ages of five to fourteen years for six months, whereas those >15 years are recommended to receive INH for 12 months. Cotrimoxazole is given to adolescents with a CD4 count below 200 cells/mm^3^ and is discontinued once CD4 is above 200 cells/mm^3^ [18]. Routine care includes TB screening if symptomatic with a CXR, sputum for Xpert MTB/RIF and culture, at the discretion of the primary physician. All participants received the SA standard Expanded Program on Immunization (EPI) including BCG at birth, diphtheria‐pertussis‐tetanus (DwPT), *Haemophilus influenzae* type b (HIB), hepatitis B (HBV), measles and polio immunizations [19]. Notably, as acellular pertussis and pneumococcal conjugate vaccines were only introduced into the public immunization programme in 2009, no participant would have received these vaccines.

### 2.2 Laboratory investigations

QFT tests results were expressed as international units (IU/mL) and considered positive if Nil value was ≤0.8 UL/mL, the TB Ag was ≥0.35 IU/mL and <25 % of Nil value after subtraction of the negative control value (Ag‐Nil) and the Mitogen‐Nil value was ≥0.5 IU/mL; Negative if the Nil value was ≤0.8 IU/mL, the TB Ag was <0.35 IU/mL or ≤0.35 and <25 % of Nil value after subtraction of the negative control value (Ag‐Nil) and the Mitogen‐Nil value was ≥0.5 IU/mL. For indeterminate status, the Nil value was ≤0.8 UL/mL, the TB Ag was <0.35 IU/mL or ≤0.35 and <25 % of Nil value after subtraction of the negative control value (Ag‐Nil) and the Mitogen‐Nil value was <0.5 IU/mL or if the Nil value was <0.8 UL/mL.

### 2.3 Diagnosis of TB

Diagnoses were obtained from study laboratory results as well as data abstracted from a provincial database, that recorded initiation of TB treatment [20]. We included all TB diagnoses between study enrolment and follow‐up to 31^st^ October 2018. A paediatric infectious disease specialist reviewed all records and diagnoses in the participant’s clinical record. TB disease was defined as “confirmed” (culture‐confirmed or Xpert MTB/RIF positive) or “unconfirmed” (participants who presented with clinical signs and symptoms suggestive of TB and documented as initiating TB treatment at their primary care facilities, but without microbiological confirmation) [21].

### 2.4 Statistical methods

Data analysis used Stata version 14.1 (StataCorp, College Station, Texas); all statistical tests were two‐sided at α = 0.05. The primary outcomes were the incidence of a positive QFT and of TB disease over time. Following standard data exploration, regression analyses were structured as follows: (i) Prevalence of QFT positivity (QFT+) at enrolment (cross‐sectional, logistic regression); (ii) QFT conversion rates during first three years (Cox proportional hazards, PH and (iii) negative binomial regression models with generalized estimating equations, GEE), restricted to those testing QFT negative at baseline, with person‐time censored at first QFT+ test or last known time alive; (iv) TB incidence rates over full follow‐up period were calculated using Cox PH for time‐to‐first TB event, person‐time staggered by six months for prevalent TB cases and censored at first TB event or last known time alive up to 31^st^ October 2018 and (v) negative binomial regression with GEE was used for overall incidence of all TB events, person‐time staggered for prevalent cases and censored at last known time alive up to 31^st^ October 2018. Loss to follow‐up was not a competing event as we could still access recorded TB episodes from a database covering all public health services in the province [20]. CD4 count, viral load, Tanner stage, BMI and age were time‐varying (annual changes); for these variables, missing data were interpolated using standard approaches, assuming data to be missing at random. Known category boundaries were assigned *a priori* based on clinical relevance. Potential third variables were identified *a priori* using a causal approach guided by the literature [22]. For all analyses comparing APHIV to HIV− youth, socioeconomic status was considered the most important potential confounder, expressed using relative measures of poverty (based on a standardized asset score and parental employment) [23]. We also adjusted for time‐varying age and Tanner staging throughout, to control for potentially differential trajectories of adolescent development between HIV+ and HIV− youth. We explored the impact of HIV viral suppression on differential risks of TB infection (QFT +) and TB disease between APHIV and HIV− youth by creating four dummy variables to compare each category of HIV‐VL suppression (at 40 and 1000 copies/mL) separately to the reference group of HIV− adolescents. Sensitivity analyses evaluated differences by confirmed TB diagnosis, variation in person‐time definition and without interpolation of missing data points.

Ethical approval was given by the University of Cape Town and Stellenbosch University. A parent or legal guardian provided informed consent and assent was obtained from all adolescents; this was renewed annually. All participants knew their HIV status as a pre‐requisite to study enrolment and gave permission for medical record reviews.

## 3 Results

**Figure 1:**
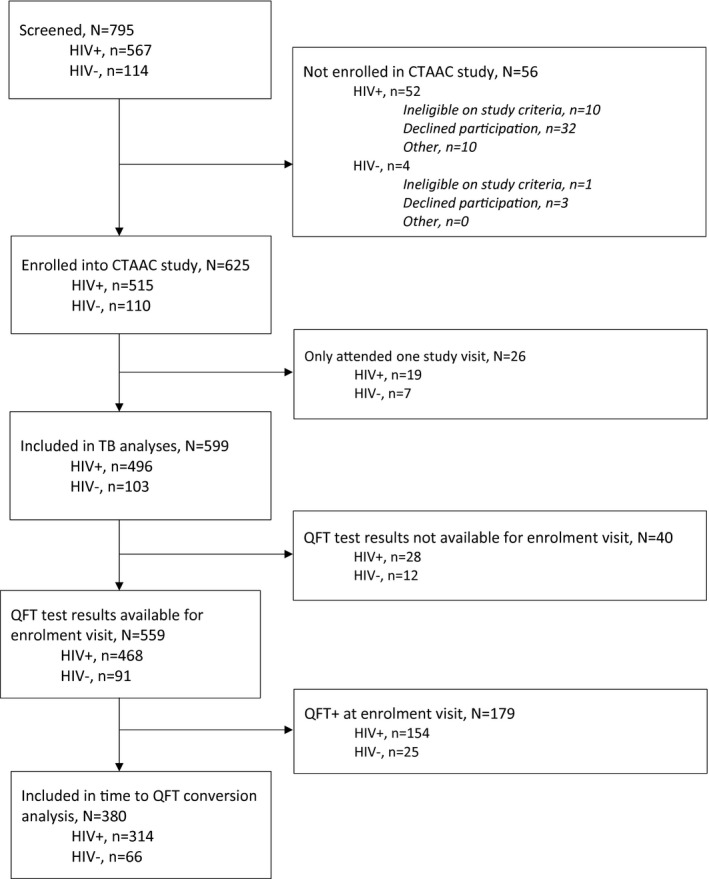
Study flow diagram.

Of 795 eligible adolescents screened, 625 (515 APHIV, 110 HIV−) were enrolled (Figure 1). Most study participants attended at least seven study visits over the follow‐up period (383 [74%] of 515 APHIV; and 85 [77%] of 110 HIV−). Data collected at two or more visits were available for 599 adolescents (496 [96%] of APHIV, 103 [94%] of HIV−; *p* = 0.20), who were followed for a median of 3.1 years (interquartile range, IQR 3.0 to 3.4; APHIV for 3.1 years and HIV− for 3.4 years). There were two deaths (both APHIV, one suicide and one who died at home after disengaging from clinical care). Enrolment characteristics are shown in Table 1; minor differences between those included in the analysis versus those excluded are shown in Table S1.

**Table 1:** Characteristics of study participants at enrolment

Characteristic	Total (N = 599)	HIV+ (N = 496)	HIV− (N = 103)	*p*‐value
Age (years)	12.0 (10.6 to 13.3)	12.0 (10.7 to 13.3)	11.6 (10.0 to 13.4)	0.199
Male sex	298 (50%)	251 (51%)	47 (46%)	0.358
Relative poverty categories^1^				
Least disadvantaged	209 (35%)	191 (39%)	18 (18%)	<0.001
Moderate disadvantage	203 (34%)	179 (36%)	24 (24%)	
Most disadvantaged	185 (31%)	125 (25%)	60 (59%)	
Previous TB disease	301 (51%)	298 (61%)	3 (3%)	<0.001
On treatment for any TB disease at enrolment	7 (1%)	7 (1%)	0	0.225
Previous isoniazid preventive therapy	134 (22%)	131 (27%)	3 (3%)	<0.001
Current known TB contact	13/555 (2%)	9/459 (2%)	4/96 (4%)	0.194
Tanner stage				
Prepubertal (Stage I)	265 (44%)	231 (47%)	34 (33%)	0.041
Adolescent (Stages II‐IV)	303 (51%)	240 (48%)	63 (61%)	
Mature (Stage V)	31 (5%)	25 (5%)	6 (6%)	
Body mass index (BMI, (kg/m^2^)	17.3 (16.1 to 19.2)	17.1 (16.0 to 18.9)	18.7 (16.6 to 21.5)	0.001
BMI categories				
Underweight, BMI < 18.5	397 (66%)	347 (70%)	50 (48%)	<0.001
Normal, BMI ≥ 18.5 < 25	179 (30%)	134 (27%)	45 (44%)	
Overweight/obese, BMI ≥ 25	23 (4%)	15 (3%)	8 (8%)	
Age at ART initiation (years)	n/a	4.4 (2.0 to 7.6)	n/a	n/a
Categories of age at ART initiation				
≤2 years	n/a	120 (25%)	n/a	n/a
>2, <6 years	n/a	196 (40%)	n/a	n/a
≥6 years	n/a	172 (35%)	n/a	n/a
HIV viral load (log_10_ copies/mL)	n/a	1.59 (1.59 to 1.60)	n/a	n/a
Categories of HIV viral load				
<40 copies/mL	n/a	376 (76%)	n/a	n/a
40 to 1000 copies/mL	n/a	56 (11%)	n/a	n/a
≥1000 copies/mL	n/a	64 (13%)	n/a	n/a
CD4 cell count (cells/µL)	n/a	713 (564 to 954)	n/a	n/a
CD4 cell count categories				
>500 cells/µL	n/a	414 (83%)	n/a	n/a
>350, ≤500 cells/µL	n/a	39 (8%)	n/a	n/a
≤350 cells/µL	n/a	43 (9%)	n/a	n/a
QFT result at enrolment^2^				
QFT positive	179 (30%)	154 (31%)	25 (24%)	0.052
QFT negative	362 (60%)	299 (60%)	63 (61%)	
QFT indeterminate	18 (3%)	15 (3%)	3 (3%)	
QFT results not available	40 (7%)	28 (6%)	12 (12%)	

Numbers are median (interquartile range) or n (column percentage); *p*‐values from Chi2 or Kruskal–Wallis testing, not corrected for multiplicity. QFT, interferon gamma release assay (QuantiFERON‐TB).

^1^ Tertiles of a continuous score incorporating a standardized asset score (including type of housing, access to running water and flush toilet), employment and education; missing (n = 2)

^2^ the percentage of QFT‐positive participants differs from the number quoted in the manuscript as the denominator in Table 1 is different and excludes 40 participants who did not have a QFT result available from their enrolment visit.

Overall, 50% (298/599) were male, with median enrolment age of 12 (IQR 10.6 to 13.3) years, similar for APHIV and HIV− participants (Table 1). At enrolment, APHIV (vs. HIV−) were more likely to be pre‐pubertal (Tanner Stage 1: 231/496 [47%] vs. 34/103 [33%] and underweight (BMI < 18.5 in 347/496 [70%] vs. 50/103 [48%]). They were also more likely to have a prior history of TB disease (298/496 [61%] vs. 3 [0.3%]). APHIV participants had initiated ART at median age 4.4 (IQR 2.1 to 7.6) years, with 376/496 (76%) having an HIV viral load (VL) <40 copies HIV RNA copies/mm^3^; median CD4 count was 713 (IQR: 564 to 954) cells/mm^3^ at enrolment (Table 1). Median ART duration was 7.6 years (IQR: 4.6 to 9.2). Two hundred and seventy (54%) adolescents were on an efavirenz‐based regimen and 143 (29%) were on a lopinavir/ritonavir‐based regimen at enrolment, whereas 83 (17%) were on other regimens. One hundred and thirty‐one (27%) APHIV and 3 (3%) HIV− adolescents had received prior INH prophylaxis and 9/459, 2% of APHIV and 4/96 (4%) HIV− adolescents had a household TB contact at enrolment.

### 3.1 QFT positivity

At enrolment, 179 (32%) of 559 adolescents were QFT+, with no difference by HIV status (APHIV 154/468, 33%; HIV− 25/91, 27%; *p* = 0.31; Table 1). There was also no difference in the number of indeterminate tests by HIV status. Among APHIV, concurrent diagnosis of TB disease was associated with increased odds of QFT+ (vs. those without TB at enrolment, OR 5.23, 95% CI 1.00 to 27.30; *p* = 0.049; data not shown). No other enrolment characteristics predicted baseline prevalence of QFT+ among either APHIV or HIV− youth (Table S2), after excluding those with prevalent TB disease. By 36 months of follow‐up, the cumulative prevalence of QFT+ was 46% (95% CI 42% to 50%) among 590 participants with known QFT results; prevalence was similar for APHIV (225/492, 46%; 95%CI 41% to 50%) and HIV− adolescents (44/98, 45%; 95% CI 35% to 55%), *p* = 0.88.

**Figure 2:**
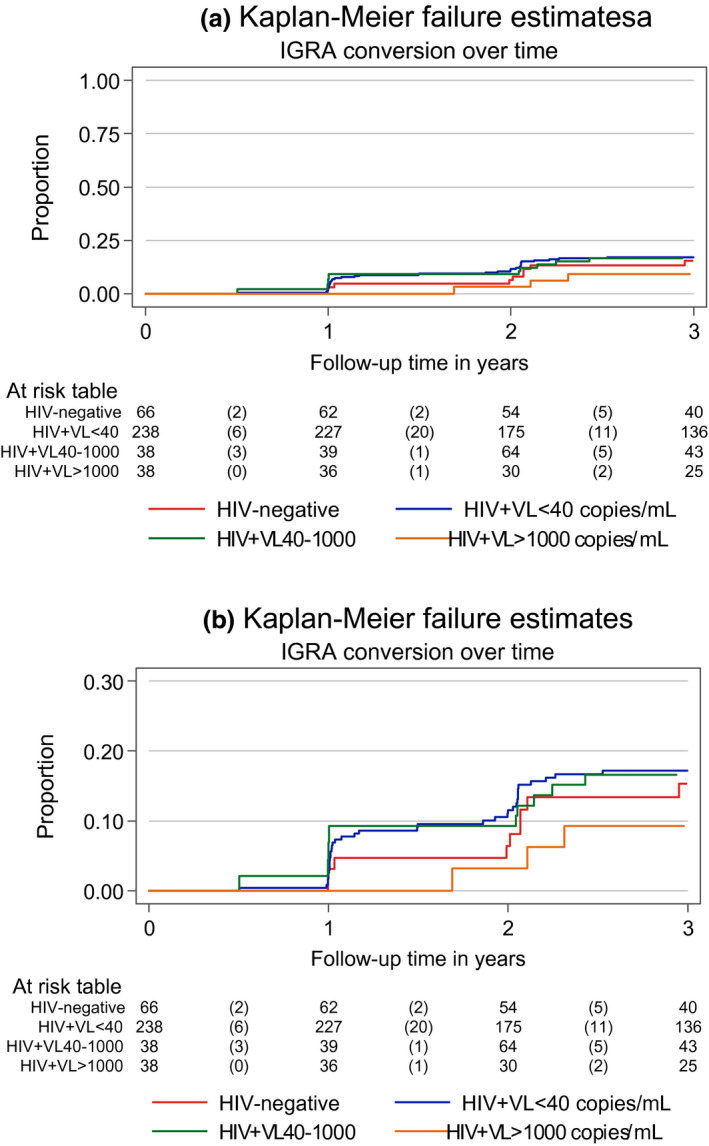
Kaplan‐Meier estimates for QFT conversion among APHIV and HIV‐negative youth over the first 36 months of follow‐up, stratified by time‐varying HIV viral load: (a) overall and (b) with restricted Y‐axis.

**Figure 3:**
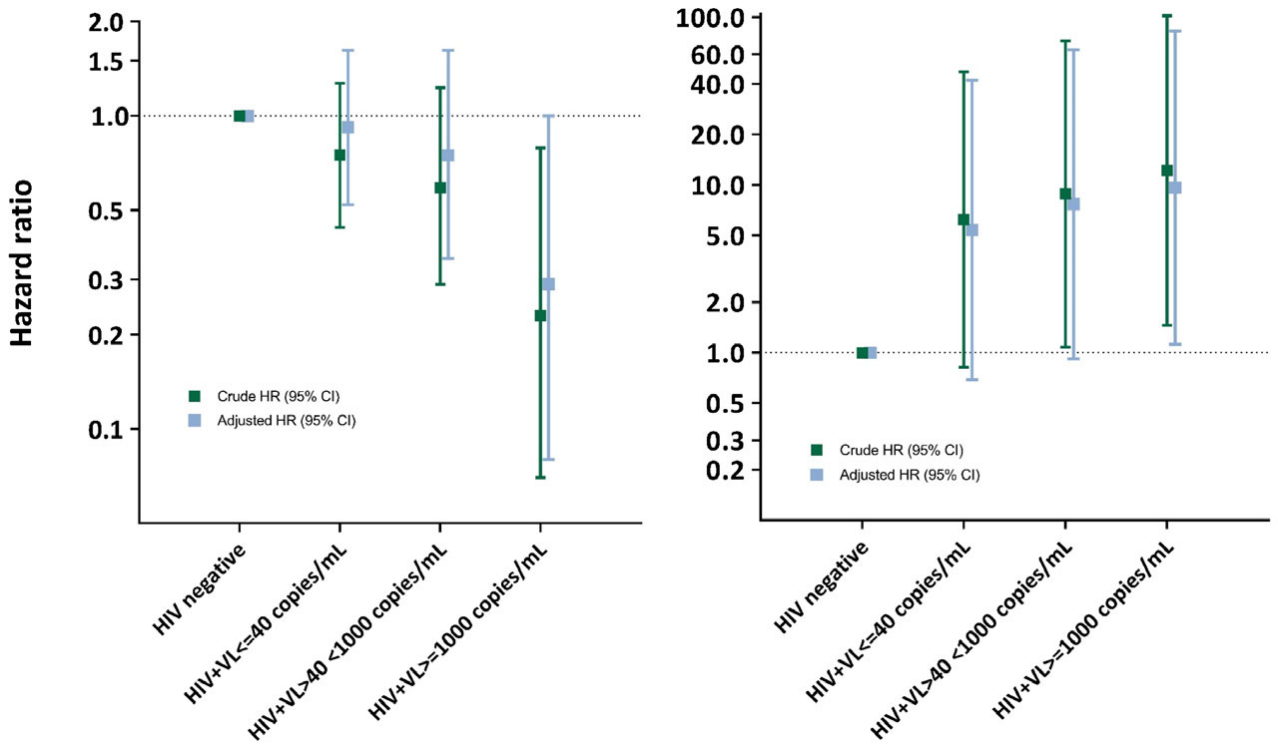
Relative hazard of (a) QFT conversion and (b) incident tuberculosis disease comparing APHIV to HIV‐negative youth in strata of HIV viral control during follow‐up.

Among 380 adolescents initially QFT negative, 82 subsequently tested QFT+ (63/314 APHIV and 19/66 HIV−), Table S3. In this group of adolescents, a total of 1110 QFT tests (mean of 3 tests per individual) were conducted during the first three years of study follow‐up, with median 12.4 months (IQR 12.0 to 14.0) between consecutive tests. The overall QFT conversion incidence was 28.8/person‐year (PY); 26.7/PY (95% CI 20.8 to 34.1) among APHIV and 39.1/PY (95% CI 24.9 to 61.2) among HIV− adolescents. (Table S3). Using a time‐to‐event approach, APHIV had marginally lower hazard of QFT conversion than HIV− (HR 0.65, 95% CI 0.39 to 1.09), Table 2a. Other factors associated with QFT conversion overall are shown in Table 2a; and restricted to APHIV, in Table 2b. There was a notable gradient in hazard of QFT conversion by CD4 count and VL, (Table 2a, Table 2b; Figures 2a,b and 3a), in both crude and adjusted analyses. Compared with all HIV− youth, APHIV with VL ≤ 40 copies/mL had 25% lower hazard of QFT conversion (HR 0.75, 95% CI 0.44 to 1.27). Those with VL > 40 but <1000 copies/mL had an even lower relative hazard of QFT conversion (HR 0.59, 95% CI 0.29 to 1.23), whereas the lowest relative hazard of QFT conversion was seen among APHIV with VL ≥ 1000 copies/mL (vs. all HIV− youth, HR 0.23; 95% CI 0.08 to 1.00, *p* = 0.05; Figure 3a).

**Table 2a:** Time to QFT conversion among previously QFT‐negative study participants, comparing APHIV to HIV‐youth (N = 380): crude and adjusted hazard ratios from Cox proportional hazards regression

	Crude HR (95% CI)	aHR (95% CI)^1^
HIV infection: HIV positive versus HIV negative	0.65 (0.39 to 1.09)	–
Effects of time‐varying HIV viral suppression on relative Hazard of QFT conversion among HIV+ versus HIV− youth		
HIV negative (reference)	1.00	1.00
HIV positive, viral load ≤40 copies/mL	0.75 (0.44 to 1.27)	0.92 (0.52 to 1.62)
HIV positive, viral load >40, <1000 copies/mL	0.59 (0.29 to 1.23)	0.75 (0.35 to 1.62)
HIV positive, viral load ≥1000 copies/mL	**0.23 (0.07 to 0.79)**	**0.29 (0.08 to 1.00)**
Male versus female sex	0.71 (0.46 to 1.11)	–
Age at study visit (time‐varying, per year increase)	0.92 (0.81 to 1.05)	**0.86 (0.73 to 1.00)**
Categories of age at study visit (time‐varying, in years)		
<12 years (reference)	1.00	–
≥12, <14 years	1.55 (0.73 to 3.31)	–
≥14, <16 years	1.14 (0.52 to 2.52)	–
≥16 years	1.00 (0.39 to 2.56)	–
BMI categories (time‐varying)		
Normal weight, ≥18, <25 kg/m^2^ (reference)	1.00	–
Underweight, <18 kg/m^2^	0.67 (0.41 to 1.10)	–
Overweight/obese, ≥25 kg/m^2^	0.87 (0.44 to 1.72)	–
Tanner staging at study visit (time‐varying)		
Stage I (pre‐adolescent), reference	1.00	1.00
Stage II to IV (adolescent)	1.02 (0.56 to 1.84)	1.34 (0.71 to 2.52)
Stage V (mature)	1.46 (0.73 to 2.90)	**2.40 (1.06 to 5.42)**
Known TB contact at enrolment (yes vs. no)	1.78 (0.33 to 5.65)	–

aHR, adjusted hazard ratio; CD, cluster of differentiation; CI, confidence intervals; HIV, human immunodeficiency virus; HR, hazard ratio; QFT, interferon gamma release assay (QuantiFERON‐TB).

Bold indicates statistically significant values.

^1^ Multivariable model adjusted for all variables with adjusted estimates shown as well as baseline measures of relative poverty.

**Table 2b:** Time to QFT conversion among previously QFT‐negative study participants, restricted to APHIV (N = 314): crude and adjusted hazard ratios from Cox proportional hazards regression

	Crude HR (95% CI)	aHR (95% CI)^1^
HIV viral load log_10_ copies/mL (per log_10_ increase)	**0.58 (0.36 to 0.95)**	0.67 (0.40 to 1.10)
CD4 cell count categories		
HIV positive, CD4 ≥ 500 (reference)	1.00	1.00
HIV positive, CD4 < 500	**0.28 (0.11 to 0.71)**	**0.38 (0.15 o 0.97)**
CD4 cell count cells/µL (per 100 increase)	**1.08 (1.00 to 1.17)**	
Male versus female sex	0.74 (0.45 to 1.23)	–
Age at study visit (time‐varying, per year increase)	0.90 (0.77 to 1.05)	0.84 (0.70 to 1.01)
Categories of age at study visit (time‐varying, in years)		
<12 years (reference)	1.00	–
≥12, <14 years	1.13 (0.52 to 2.48)	–
≥14, <16 years	0.93 (0.41 to 2.10)	–
≥16 years	0.76 (0.27 to 2.14)	–
BMI categories (time‐varying)		
Normal weight, ≥18, <25 kg/m^2^ (reference)	1.00	–
Underweight, <18 kg/m^2^	0.65 (0.38 to 1.11)	–
Overweight/obese, ≥25 kg/m^2^	0.68 (0.26 to 1.73)	–
Tanner staging at study visit (time‐varying)		
Stage I (pre‐adolescent), reference	1.00	1.00
Stage II to IV (adolescent)	0.99 (0.51 to 1.89)	1.39 (0.69 to 2.79)
Stage V (mature)	1.51 (0.70 to 3.24)	**2.51 (1.01 to 6.26)**
Known TB contact at enrolment (yes vs. no)	0.76 (0.10 to 5.50)	–
Age at ART initiation		
≤2 years (reference)	1.00	–
>2, <6 years	1.08 (0.55 to 2.11)	–
≥6 years	1.10 (0.56 to 2.17)	–

aHR, adjusted hazard ratio; CD, cluster of differentiation; CI, confidence intervals; HIV, human immunodeficiency virus; HR, hazard ratio; QFT, interferon gamma release assay (QuantiFERON‐TB).

Bold indicates statistically significant values.

^1^ Multivariable model adjusted for all variables with adjusted estimates shown as well as baseline measures of relative poverty.

### 3.2 TB disease

At enrolment, seven (APHIV) participants had TB disease; (5 cases were confirmed TB). During follow‐up, an additional 35 episodes of incident TB (20 confirmed (19 APHIV, 1 HIV−), 15 unconfirmed) occurred in 31 participants (two APHIV had two episodes, and one had three). During follow‐up, there were 30 cases of pulmonary TB (PTB), two cases of TB meningitis (one APHIV and one HIV− participant) and three cases of unspecified TB. A total of 14 (3 %) APHIV and 1 (1%) HIV− participants were hospitalized for TB. In total, 11 CXR results were reported by radiologists of which seven were reported as suggestive of PTB. All *Mtb* isolates were sensitive, except for one that was INH mono‐resistant.

The overall incidence of TB disease was 1.9/100PY (95% CI 1.4 to 2.6) for the full cohort (Table S7). APHIV had a markedly higher incidence than HIV− participants in both crude and adjusted analysis (2.2/100PY, 95% CI 1.6 to 3.1 vs. 0.3/100PY, 95% CI 0.04 to 2.2; IRR 7.36, 95% CI 1.01 to 53.55; Table S7a). When restricting TB diagnoses to those with confirmed TB disease, TB incidence rate for the full cohort was 1.1/100PY (Table S4) with a consistently higher incidence in APHIV (1.3/100PY) than HIV− (0.3/100PY).

CD4 count and VL were strong predictors of overall incidence of TB disease (confirmed and unconfirmed) in APHIV. APHIV with VL > 1000 copies/mL had 12‐fold higher hazard of TB compared to all HIV− youth (HR 12.21, 95% CI 1.46 to 102.36, Table 3a; in contrast, those with VL < 40 copies/mL had only a slightly higher hazard relative to HIV‐youth (HR 6.22, 95% CI 0.82 to 47.35). The hazard for APHIV with VL 40 to 1000 copies/mL (vs. HIV− youth) fell in between the former two categories (HR 8.84, 95% CI 1.08 to 72.38); inferences were unchanged after adjustment (Table 3a and Figure 3a). A similar gradient was observed comparing strata of CD4 cell counts (Table 3a,b). Increasing VL or decreasing CD4 cell count was therefore associated with increased likelihood of TB disease but a decreased likelihood of QFT+ conversion. Figure 3a,b provide a direct visual comparison of changing relative hazard by HIV disease severity. Although these associations were attenuated when restricting events to only confirmed TB diagnoses, the pattern of a risk gradient by disease severity remained (Tables S5 and S6).

**Table 3a:** Time to first TB disease event comparing APHIV to HIV‐negative youth (N = 599): crude and adjusted hazard ratios from Cox proportional hazards regression

	Crude HR (95% CI)	aHR (95% CI)^1^
HIV infection: HIV positive versus HIV negative	**7.56 (1.02 to 56.04)**	–
Effects of time‐varying HIV viral suppression on relative hazard of incident TB among HIV+ versus HIV− youth		
HIV negative (reference)	1.00	1.00
HIV positive, viral load ≤ 40 copies/mL	6.22 (0.82 to 47.35)	5.39 (0.69 to 42.17)
HIV positive, viral load > 40, <1000 copies/mL	**8.84 (1.08 to 72.38)**	7.69 (0.92 to 64.14)
HIV positive, viral load ≥ 1000 copies/mL	**12.21 (1.46 to 102.26)**	**9.65 (1.12 to 82.92)**
Male versus female sex	0.84 (0.41 to 1.70)	–
Age at study visit (time‐varying, per year older)	**1.35 (1.08 to 1.68)**	**1.44 (1.12 to 1.86)**
Categories of age at study visit (time‐varying)		
<12 years (reference)	1.00	–
≥12, <14 years	3.96 (0.48 to 32.42)	–
≥14, <16 years	5.26 (0.65 to 42.88)	–
≥16 years	6.94 (0.77 to 62.74)	–
BMI categories (time‐varying)		
Normal weight, ≥ 18, <25 kg/m^2^ (reference)	1.00	–
Underweight, <18 kg/m^2^	1.29 (0.61 to 2.72)	–
Overweight/obese, ≥25 kg/m^2^	0.33 (0.04 to 2.52)	–
Tanner staging at study visit (time‐varying)		
Stage I (reference)	1.00	1.00
Stage II to IV	1.20 (0.43 to 3.33)	0.70 (0.23 to 2.09)
Stage V	1.15 (0.33 to 4.02)	0.48 (0.12 to 1.95)
Known TB contact at enrolment (yes vs. no)	1.45 (0.28 to 7.43)	–
QFT result at study enrolment		
QFT negative (reference)	1.00	–
QFT positive	0.64 (0.26 to 1.59)	–
QFT test results unknown	1.20 (0.28 to 5.18)	–

aHR, adjusted hazard ratio; CD, cluster of differentiation; CI, confidence intervals; HIV, human immunodeficiency virus; HR, hazard ratio; QFT, interferon gamma release assay (QuantiFERON‐TB).

Bold indicates statistically significant values.

^1^ Multivariable model adjusted for all variables with adjusted estimates shown as well as baseline measures of relative poverty.

**Table 3b:** Factors associated with time to first TB disease event among APHIV (N = 496): crude and adjusted hazard ratios from Cox proportional hazards regression

	Crude HR (95% CI)	aHR (95% CI)^1^
HIV viral load log_10_ copies/mL (per log_10_ increase)	**1.41 (1.00 to 1.98)**	1.25 (0.86 to 1.82)
HIV viral suppression categories (time‐varying)		
HIV viral load ≤40 copies/mL (reference)	1.00	–
HIV viral load >40, <1000 copies/mL	1.41 (0.59 to 3.40)	–
HIV viral load ≥1000 copies/mL	1.96 (0.76 to 5.04)	–
CD4 cell count cells/µL (per 100 increase)	0.90 (0.79 to 1.03)	–
CD4 cell count categories (time‐varying)		
CD4 ≥ 500 cells/µL (reference)	1.00	1.00
CD4 < 500 cells/µL	**2.35 (1.10 to 5.03)**	1.74 (0.75 to 4.03)
Male versus female sex	0.82 (0.40 to 1.69)	–
Age at study visit (time‐varying, per year increase)	**1.33 (1.06 to 1.68)**	**1.37 (1.06 to 1.78)**
BMI categories (time‐varying)		
Normal weight, ≥18, <25 kg/m^2^ (reference)	1.00	–
Underweight, <18 kg/m^2^	1.35 (0.63 to 2.91)	–
Overweight/obese, ≥25 kg/m^2^	0.44 (0.06 to 3.42)	–
Tanner staging at study visit (time‐varying)		
Stage I (reference)	1.00	1.00
Stage II to IV	1.20 (0.43 to 3.36)	0.74 (0.25 to 2.21)
Stage V	1.35 (0.39 to 4.72)	0.59 (0.14 to 2.44)
Known TB contact at enrolment (yes vs. no)	1.93 (0.34 to 10.85)	–
QFT result at study enrolment		
QFT negative (reference)	1.00	–
QFT positive	0.64 (0.26 to 1.60)	–
QFT test results unknown	1.66 (0.38 to 7.22)	–
Age at ART initiation		
≤2 years (reference)	1.00	–
>2, <6 years	3.50 (0.77 to 15.80)	–
≥6 years	**5.68 (1.30 to 24.87)**	–

aHR, adjusted hazard ratio; CD, cluster of differentiation; CI, confidence intervals; HIV, human immunodeficiency virus; HR, hazard ratio; QFT, interferon gamma release assay (QuantiFERON‐TB).

Bold indicates statistically significant values.

^1^ Multivariable model adjusted for all variables with adjusted estimates shown as well as baseline measures of relative poverty.

Of 28 APHIV with a single incident episode of TB disease, 16 (57%) completed treatment and 8 (29%) had weight gain and resolution of symptoms but we could not confirm treatment completion. One (3.6%) participant had treatment stopped after the TB diagnosis was changed after extensive workup, whereas a further two (7.1%) participants were lost to follow‐up after TB diagnosis and no further information could be found. One APHIV had three episodes of TB disease and did not complete treatment during the first episode. This participant completed treatment for the subsequent two episodes. Of the two participants with two incident TB disease episodes, one completed treatment for both episodes (with a negative culture between episodes) and the other completed treatment for the first episode but was lost to follow‐up after diagnosis of the second episode. The HIV‐negative participant completed treatment and had resolution of symptoms.

## 4 Discussion

These data demonstrate that, in the context of high HIV and TB burden, APHIV have a much higher rate of TB disease than HIV− adolescents despite ART and despite similar rates of QFT conversion. These findings appeared driven mainly by incomplete CD4 immune reconstitution or non‐suppressive ART, as evidenced by higher rates of TB disease in those with lower CD4 counts or higher VL. In addition, the prevalence of TB infection was high in both APHIV and HIV− participants. These findings highlight that *M.tb* infection is common and that TB remains an important cause of morbidity among APHIV.

The prevalence of positive QFT at enrolment for the entire cohort was 32% with a cumulative prevalence of 46% after three years of follow‐up at a median age of 15 years. This is similar to the prevalence reported in Western Kenya where at 14.4 years the prevalence of TB infection (using Tuberculin skin testing) was also 32%; however, only 0.5% adolescents were living with HIV in that study and 41% had unknown HIV status [14]. The prevalence in our study is similar to the 53% prevalence reported in Cape Town in 2011 in adolescents between 14 and 17 years [13]. However, in our study, a positive QFT was not predictive of developing TB. In addition, low CD4 count or high VL were associated with decreased risk of QFT conversion. This is consistent with an adult study that reported a moderate predictive effect of QFT to diagnose incident TB, with a negative effect of CD4 count on sensitivity [24].

The incidence of TB in APHIV in our study was very high, at 2.2/100 PY. This is higher than the rate of 0.7/100 PY reported from the same setting in 2011 (in both APHIV and HIV− adolescents) and higher than in Ethiopian adolescents (1.6/100 PY) on ART for more than five years [25]. This may be due to the extremely high prevalence of TB in Western Cape communities where most of these adolescents live or high exposure in HIV households [26]. To our knowledge, this is the first study from sub Saharan Africa to report on the incidence of confirmed TB in APHIV. The rate of confirmed TB was 1.3 (CI: 0.80 to 1.97)/100 PY which is very high, especially given that adolescents were on ART for several years. There are no comparable studies of APHIV, but between 2005 and 2007 an adolescent cohort in Western Cape, South Africa reported TB disease incidence of 0.45 per 100 person years, however, this study did not include routine HIV testing. Low CD4 count and high VL were associated with increased risk of TB. The cohort had relatively high rates of immune reconstitution as well as viral load suppression and therefore the incidence of TB may be higher in other settings where the majority of adolescents do not have well‐controlled HIV. This highlights a vulnerable group of APHIV who are at increased risk of TB and who may paradoxically have false‐negative QFT results. The results also emphasise the need to integrate HIV and TB adolescent programmes.

### 4.1 Limitations and strengths

Limitations include that history of prior TB and TB contact could not always be verified. Data on INH prophylaxis were limited. Serial QFT testing was not done on QFT‐positive participants, so QFT reversion could not be reported. There was limited availability of CXRs from the time of TB diagnosis. An additional limitation is that we only compared APHIV with those that were HIV−. Adolescents living with horizontally acquired HIV may have a different incidence of TB disease. Strengths of our study include long‐term follow‐up of a large cohort of APHIV in a resource‐limited setting with high cohort retention; microbiologic confirmation of TB and longitudinal QFT testing over an extended period of time. In addition a comparison group of matched HIV− participants strengthens inferences by providing insights into the background risks of TB infection and disease in this setting.

## 5 Conclusions

In summary, we found that despite similar rates of *Mtb* infection, there is a higher rate of TB disease in APHIV on ART compared to HIV− adolescents. This increased rate of TB disease was driven by high viral loads and decreased CD4 counts, highlighting the importance of screening for incident TB disease in APHIV failing ART. These adolescents should have access to clinical assessment including CXR and rapid molecular diagnostics and culture as QFT may not be sensitive or predictive of TB disease. In addition, strategies to prevent TB disease such as TB preventive therapy as well as strategies to enhance treatment adherence should be strengthened.

## Competing interest

The authors have no conflict of interest to declare.

## Authors’ contributions

LJF (corresponding author) was the study doctor, assisted with collection of data; helped in planning the analyses; wrote the first draft of the manuscript and confirms that she had full access to all the data and takes final responsibility for the decision to submit for publication. KB was responsible for data management and oversight. SLR did the analysis with input from LJF. LM and HJZ conceived the CTAAC study, and were responsible for study design, funding, implementation and overall leadership. KW, WP, SR and SB were responsible for QFT testing on the study. LG was a study doctor and contributed to the initial study design. All authors contributed to and approved the final manuscript.

## Supporting information


**Table S1**. Characteristics of APHIV and HIV‐negative youth at study enrolment, comparing those included vs excluded from analysis
**Table S2**. Factors associated with QFT positivity at enrolment, among APHIV who did not have TB disease at baseline (N = 461): odds ratios from logistic regression analysis
**Table S3.** Incidence rates of QFT conversion over the first three years of follow‐up (among study participants who tested QFT negative at enrolment)
**Table S4**. Sensitivity analysis I: variation in incidence of tuberculosis disease (TB) overall and by HIV status, using varying definitions of TB events and duration of person‐time
**Table S5**. Sensitivity analysis II: TB occurrence and predictors among APHIV: all TB diagnoses versus bacteriologically confirmed TB diagnoses only, using negative binomial regression with GEE to estimate overall TB incidence, including recurrent events [incidence rate ratios]
**Table S6**. Sensitivity analysis III: Predictors of time to first TB event among HIV+ youth: all TB diagnoses versus bacteriologically confirmed TB diagnoses only, using Cox proportional hazards regression
**Table S7a**. Incidence rates of all tuberculosis disease events over follow‐up, comparing APHIV to HIV+ youth: crude and adjusted incidence rate ratios from negative binomial regression with generalized estimating equations
**Table S7b**. Incidence of all tuberculosis disease events among APHIV, stratified by participant characteristics: crude and adjusted incidence rate ratios from negative binomial regression with generalized estimating equations

## References

[1] Marais B , Gie R , Schaaf H , Hesseling AC , Obihara CC , Starke JJ , et al. The natural history of childhood intra‐thoracic tuberculosis: a critical review of literature from the pre‐chemotherapy era [State of the Art]. Int J Tuberc Lung Dis. 2004;8(4):392–402. 15141729

[2] Dodd PJ , Looker C , Plumb ID , Bond V , Schaap A , Shanaube K , et al. Age‐ and sex‐specific social contact patterns and incidence of mycobacterium tuberculosis infection. Am J Epidemiol. 2016;183(2):156–66. 26646292 10.1093/aje/kwv160 PMC4706676

[3] Snow KJ , Sismanidis C , Denholm J , Sawyer SM , Graham SM . The incidence of tuberculosis among adolescents and young adults: a global estimate. Eur Respir J. 2018;51:1702352. 29467206 10.1183/13993003.02352-2017

[4] Comstock GW , Livesay VT , Woolpert SF . The prognosis of a positive tuberculin reaction in childhood and adolescence. Am J Epidemiol. 1974;99(2):131–8. 4810628 10.1093/oxfordjournals.aje.a121593

[5] Marais BJ , Gie RP , Schaaf HS , Hesseling AC , Obihara CC , Nelson LJ , et al. The clinical epidemiology of childhood pulmonary tuberculosis: a critical review of literature from the pre‐chemotherapy era [State of the Art]. Int J Tuberc Lung Dis. 2004;8(3):278–85. 15139465

[6] Lawn SD , Bekker L‐G , Middelkoop K , Myer L , Wood R . Impact of HIV infection on the epidemiology of tuberculosis in a peri‐urban community in South Africa: the need for age‐specific interventions. Clin Infect Dis. 2006;42(7):1040–7. 16511773 10.1086/501018

[7] Lawn SD , Bekker L‐G , Wood R . How effectively does HAART restore immune responses to Mycobacterium tuberculosis? Implications for tuberculosis control. AIDS. 2005;19(11):1113–24. 15990564 10.1097/01.aids.0000176211.08581.5a

[8] Abuogi L , Mwachari C , Leslie H , Shade SB , Otieno J , Yienya N , et al. Impact of expanded antiretroviral use on incidence and prevalence of tuberculosis in children with HIV in Kenya. Int J Tuberc Lung Dis. 2013;17(10):1291–7. 24025380 10.5588/ijtld.12.0740 PMC5454479

[9] Dodd PJ , Prendergast AJ , Beecroft C , Kampmann B , Seddon JA . The impact of HIV and antiretroviral therapy on TB risk in children: a systematic review and meta‐analysis. Thorax. 2017;72(6):559–75. 28115682 10.1136/thoraxjnl-2016-209421 PMC5520282

[10] Lawn SD , Wood R . Incidence of tuberculosis during highly active antiretroviral therapy in high‐income and low‐income countries. Clin Infect Dis. 2005;41(12):1783–6. 16288404 10.1086/498308

[11] Ferrand RA , Bandason T , Musvaire P , Larke N , Nathoo K , Mujuru H , et al. Causes of acute hospitalization in adolescence: burden and spectrum of HIV‐related morbidity in a country with an early‐onset and severe HIV epidemic: a prospective survey. PLoS Med. 2010;7:e1000178. 20126383 10.1371/journal.pmed.1000178 PMC2814826

[12] Githinji LN , Gray DM , Hlengwa S , Myer L , Zar HJ . Lung function in South African adolescents infected perinatally with HIV and treated long‐term with antiretroviral therapy. Ann Am Thorac Soc. 2017;14(5):722–9. 28248548 10.1513/AnnalsATS.201612-1018OC PMC5427744

[13] Mahomed H , Hawkridge T , Verver S , Abrahams D , Geiter L , Hatherill M , et al. The tuberculin skin test versus QuantiFERON TB Gold® in predicting tuberculosis disease in an adolescent cohort study in South Africa. PLoS One. 2011;6:e17984. 21479236 10.1371/journal.pone.0017984 PMC3066222

[14] Nduba V , van’t Hoog AH , De Bruijn A , Mitchell EM , Laserson K , Borgdorff M . Estimating the annual risk of infection with Mycobacterium tuberculosis among adolescents in Western Kenya in preparation for TB vaccine trials. BMC Infect Dis. 2019;19(1):682. 31375068 10.1186/s12879-019-4314-7 PMC6679456

[15] Snow KJ , Nelson LJ , Sismanidis C , Sawyer SM , Graham SM . Incidence and prevalence of bacteriologically confirmed pulmonary tuberculosis among adolescents and young adults: a systematic review. Epidemiol Infect. 2018;146(8):946–53. 29655391 10.1017/S0950268818000821 PMC9184932

[16] Bygrave H , Mtangirwa J , Ncube K , Ford N , Kranzer K , Munyaradzi D . Antiretroviral therapy outcomes among adolescents and youth in rural Zimbabwe. PLoS One. 2012;7:e52856. 23285204 10.1371/journal.pone.0052856 PMC3527625

[17] Butte NF , Garza C , De Onis M . Evaluation of the feasibility of international growth standards for school‐aged children and adolescents. J Nutri. 2007;137(1):153–7. 10.1093/jn/137.1.153 17182818

[18] NDOH . The South African Antiretroviral Therapy Guidelines. In: Health NDo, editor. Pretoria; 2013.

[19] Ngcobo N . New EPI vaccines guidelines. Pretoria, South Africa: National Department of Health; 2010. p. 1–15.

[20] Boulle A , Heekes A , Tiffin N , Smith M , Mutemaringa T , Zinyakatira N , et al. Data centre profile: the provincial health data Centre of the Western Cape Province, South Africa. Int J Popul Data Sci. 2019;4(2). 10.23889/ijpds.v4i2.1143 PMC7482518 32935043

[21] Graham SM , Ahmed T , Amanullah F , Browning R , Cardenas V , Casenghi M , et al. Evaluation of tuberculosis diagnostics in children: 1. Proposed clinical case definitions for classification of intrathoracic tuberculosis disease. Consensus from an expert panel. J Infect Dis. 2012;205 Suppl_2:S199–S208. 22448023 10.1093/infdis/jis008 PMC3334506

[22] Mahomed H , Hawkridge T , Verver S , Geiter L , Hatherill M , Abrahams DA , et al. Predictive factors for latent tuberculosis infection among adolescents in a high‐burden area in South Africa. Int J Tuberculosis Lung Dis. 2011;15(3):331–6. 21333099

[23] Brittain K , Asafu‐Agyei NA , Hoare J , Bekker L‐G , Rabie H , et al. Association of adolescent‐and caregiver‐reported antiretroviral therapy adherence with HIV viral load among perinatally‐infected South African adolescents. AIDS Behav. 2018;22(3):909–17. 29224045 10.1007/s10461-017-2004-2 PMC6620475

[24] Santin M , Munoz L , Rigau D . Interferon‐gamma release assays for the diagnosis of tuberculosis and tuberculosis infection in HIV‐infected adults: a systematic review and meta‐analysis. PLoS One. 2012;7:e32482. 22403663 10.1371/journal.pone.0032482 PMC3293815

[25] Andrews JR , Morrow C , Walensky RP , Wood R . Integrating social contact and environmental data in evaluating tuberculosis transmission in a South African township. J Infect Dis. 2014;210(4):597–603. 24610874 10.1093/infdis/jiu138 PMC4133578

[26] Mahomed H , Ehrlich R , Hawkridge T , et al. TB incidence in an adolescent cohort in South Africa. PLoS One. 2013;8:e59652. 23533639 10.1371/journal.pone.0059652 PMC3606161

