# Supplementary material for: Tuberculosis infection and disease in South African adolescents with perinatally acquired HIV on antiretroviral therapy: a cohort study
Source: J Int AIDS Soc. 2021 Mar 14;24(3):e25671. doi: 10.1002/jia2.25671 (PMC7957181; doi:10.1002/jia2.25671)
Supplement: Supplementary file 1 — Table S1. Characteristics of APHIV and HIV‐negative youth at study enrolment, comparing those included vs excluded from analysis Table S2. Factors associated with QFT positivity at enrolment, among APHIV who did not have TB disease at baseline (N = 461): odds ratios from logistic regression analysis Table S3. Incidence rates of QFT conversion over the first three years of follow‐up (among study participants who tested QFT negative at enrolment) Table S4. Sensitivity analysis I: variation in incidence of tuberculosis disease (TB) overall and by HIV status, using varying definitions of TB events and duration of person‐time Table S5. Sensitivity analysis II: TB occurrence and predictors among APHIV: all TB diagnoses versus bacteriologically confirmed TB diagnoses only, using negative binomial regression with GEE to estimate overall TB incidence, including recurrent events [incidence rate ratios] Table S6. Sensitivity analysis III: Predictors of time to first TB event among HIV+ youth: all TB diagnoses versus bacteriologically confirmed TB diagnoses only, using Cox proportional hazards regression Table S7a. Incidence rates of all tuberculosis disease events over follow‐up, comparing APHIV to HIV+ youth: crude and adjusted incidence rate ratios from negative binomial regression with generalized estimating equations Table S7b. Incidence of all tuberculosis disease events among APHIV, stratified by participant characteristics: crude and adjusted incidence rate ratios from negative binomial regression with generalized estimating equations [file JIA2-24-e25671-s001.docx]

**SUPPLEMENTAL TABLE 1. Characteristics of APHIV and HIV-negative youth at study enrolment, comparing those included vs excluded from analysis**

| **Characteristic** | **HIV-positive (N=515)** | | | **HIV-negative (N=110)** | | |
| --- | --- | --- | --- | --- | --- | --- |
|  | **In analysis (n=496)** | **Not in analysis (n=19)** | ***p*-value** | **In analysis (n=103)** | **Not in analysis (n=7)** | ***p*-value** |
| Age (years) | 12.0 (10.7-13.3) | 11.2 (10.3-12.3) | 0.18 | 11.6 (10.0-13.4) | 13.7 (10.5-14.0) | 0.30 |
| Relative poverty categories^1^ |  |  | 0.17 |  |  | 0.29 |
| *Most disadvantaged* | 125 (25%) | 8 (42%) |  | 60 (59%) | 2 (29%) |  |
| *Moderate disadvantage* | 179 (36%) | 7 (37%) |  | 24 (24%) | 3 (43%) |  |
| *Least disadvantaged* | 191 (39%) | 4 (21%) |  | 18 (18%) | 2 (28%) |  |
| Male sex | 251 (51%) | 12 (63%) | 0.28 | 47 (46%) | 2 (29%) | 0.38 |
| Tanner stage |  |  | **0.007** |  |  | 0.46 |
| *Prepubertal (Stage I)* | 231 (47%) | 15 (83%) |  | 34 (33%) | 1 (14%) |  |
| *Adolescent (Stages II-IV)* | 240 (48%) | 2 (11%) |  | 63 (61%) | 5 (71%) |  |
| *Mature (Stage V)* | 25 (5%) | 1 (6%) |  | 6 (6%) | 1 (14%) |  |
| Body mass index (BMI, (kg/m^2^) | 17.1 (16.0-18.9) | 16.7 (15.7-18.7) | 0.56 | 18.6 (16.6-21.5) | 18.8 (17.8-23.5) | 0.24 |
| Age at ART initiation (years) | 4.4 (2.1-7.6) | 4.1 (1.4-6.4) | 0.65 | - | - | - |
| ART regimen at enrolment |  |  | 0.64 |  |  |  |
| *ABC-3TC-EFV* | 239 (48%) | 10 (53%) |  | - | - | - |
| *ABC-3TC-LPV(r)* | 106 (21%) | 4 (21%) |  | - | - | - |
| *AZT-3TC-LPV(r)* | 37 (7%) | 3 (16%) |  | - | - | - |
| *d4T-3TC-EFV* | 31 (6%) | 0 |  | - | - | - |
| *Other* | 78 (16%) | 2 (10%) |  | - | - | - |
| *Unknown* | 5 (1%) | 0 |  | - | - | - |
| HIV viral load (log_10_ copies/mL) | 1.59 (1.5901.60) | 1.59 (1.59-1.60) | 0.98 | - | - | - |
| CD4 cell count (cells/µL) | 713 (564-953) | 780 (536-1222) | 0.33 | - | - | - |
| Previous TB disease | 298 (61%) | 11 (61%) | 0.91 | 3 (3%) | 0 | 0.69 |
| On treatment for TB disease at enrolment | 7 (1%) | 0 | 0.60 | 0 | 0 | - |
| Previous isoniazid preventive therapy | 131 (27%) | 6 (33%) | 0.63 | 3 (3%) | 1 (20%) | 0.06 |
| Current known TB contact | 9 (2%) | 1 (6%) | 0.30 | 4 (4%) | 1 (20%) | 0.11 |
| QFT result at enrolment |  |  | 0.33 |  |  | **0.05** |
| *QFT positive* | 154 (31%) | 9 (47%) |  | 25 (24%) | 2 (29%) |  |
| *QFT negative* | 314 (63%) | 8 (42%) |  | 66 (64%) | 2 (29%) |  |
| *QFT results unknown* | 28 (6%) | 2 (11%) |  | 12 (12%) | 3 (42%) |  |

Abbreviations: QFT, interferon gamma release assay (QuantiFERON-TB®); mL, milliliter; uL, microliter; Numbers are median (interquartile range) or n (column percentage); p-values from Chi2 or Kruskal-Wallis testing, not corrected for multiplicity

^1^Tertiles of a continuous score incorporating a standardized asset score (including type of housing, access to running water and flush toilet), employment and education

**SUPPLEMENTAL TABLE 2. Factors associated with QFT positivity at enrolment, among APHIV who did not have TB disease at baseline (N=461): odds ratios from logistic regression analysis**

|  | **OR (95% CI)** |
| --- | --- |
| Age (per year increase) | 1.08 (0.96-1.22) |
| Male vs female sex | 1.03 (0.69-1.52) |
| Known previous (vs no previous) TB disease | 0.93 (0.62-1.38) |
| Current known TB contact (vs no current contact) | 0.59 (0.12-2.89) |
| Tanner stage |  |
| *Prepubertal (Stage I)* | 1.00 |
| *Adolescent (Stages II-IV)* | 1.18 (0.79-1.77) |
| *Mature (Stage V)* | 0.75 (0.28-1.97) |
| BMI categories |  |
| *Normal, BMI ≥ 18.5 <25* | 1.00 |
| *Underweight, BMI<18.5* | 1.19 (0.76-1.87) |
| *Overweight/obese, BMI ≥ 25* | 1.33(0.42-4.23) |
| Categories of age at ART initiation |  |
| *≤ 2 years* | 1.00 |
| *>2, < 6 years* | 0.78 (0.47-1.29) |
| *≥ 6 years* | 0.79 (0.47-1.32) |
| Categories of HIV viral load |  |
| *<40 copies/mL* | 1.00 |
| *40-1000 copies/mL* | 0.49 (0.24-1.02) |
| *>=1000 copies/mL* | 1.31 (0.73-2.34) |
| CD4 cell count categories |  |
| *<250 cells/uL* | 1.00 |
| *≥250,<350 cells/uL* | 6.53 (0.72-60.05) |
| *≥350,<500 cells/uL* | 5.38 (0.62-46.50) |
| *≥500,<800 cells/uL* | 7.39 (0.95-57.32) |
| *≥800 cells/uL* | 6.82 (0.87-53.16) |

Analysis restricted to HIV+ youth who received QFT testing at baseline without prevalent TB at enrolment

Abbreviations: OR, odds ratio; aOR, adjusted odds ratio; CI, confidence interval; QFT, interferon gamma release assay (QuantiFERON-TB®); TB, tuberculosis disease; IGRA, CD, cluster of differentiation; mL, milliliter; uL, microliter; ART, triple agent antiretroviral therapy

**SUPPLEMENTAL TABLE 3. Incidence rates of QFT conversion over the first 3 years of follow-up (among study participants who tested QFT negative at enrolment)**

|  | **Number of QFT conversions** | **Person-years** | **Rate/PY (95% CI)** |
| --- | --- | --- | --- |
| **All study participants** (HIV+ and HIV-), N=380 | 82 | 2.85 | 28.8 (23.2-35.7) |
| **By HIV status** |  |  |  |
| HIV- participants, N=66 | 19 | 0.49 | 39.1 (24.9-61.2) |
| HIV+ participants, N=314 | 63 | 2.36 | 26.7 (20.8-34.1) |
| **Restricted to HIV+ participants (N=63)** |  |  |  |
| HIV viral suppression categories (time-varying) |  |  |  |
| HIV-viral load ≤ 40 copies/mL | 48 | 1.66 | 28.9 (21.8- 38.3) |
| HIV-viral load >40, <1000 copies/mL | 12 | 0.42 | 28.8 (16.3-50.7) |
| HIV-viral load ≥ 1000 copies/mL | 3 | 0.28 | 10.6 (3.4-37.7) |
| CD4 cell count (per 100 cells/uL) |  |  |  |
| CD4 cell count categories |  |  |  |
| CD4 ≥ 500 cells/uL | 58 | 1.84 | 31.4 (24.3-40.7) |
| CD4 <500 cells/uL | 5 | 0.52 | 9.6 (4.0-23.1) |
| Age at ART initiation* |  |  |  |
| ≤ 2 years | 13 | 0.54 | 24.3 (14.1-41.8) |
| >2, <6 years | 25 | 0.97 | 25.8 (17.4- 38.1) |
| ≥6 years | 23 | 0.83 | 27.7 (18.4-41.7) |
| Sex |  |  |  |
| Female | 36 | 1.18 | 30.54 (22.03-42.34) |
| Male | 27 | 1.18 | 22.8 (15.6-33.26) |
| Categories of age at study visit (time-varying, in years) |  |  |  |
| <12 years | 9 | 0.45 | 19.7 (10.4-37.8) |
| ≥ 12, <14 years | 26 | 0.88 | 29.5 (20.1-43.3) |
| ≥ 14, <16 years | 20 | 0.74 | 26.9 (17.3-41.6) |
| ≥ 16 years | 8 | 0.28 | 28.7 (14.4-57.5) |
| BMI categories (time-varying) |  |  |  |
| Normal weight, ≥ 18, <25 kg/m^2^ | 36 | 1.03 | 34.9 (25.2-48.4) |
| Underweight, <18 kg/m^2^ | 22 | 1.15 | 19.2 (12.6-29.2) |
| Overweight/obese, ≥ 25 kg/m^2^ | 5 | 0.18 | 27.0 (11.2-64.9) |
| Tanner staging at study visit (time-varying) |  |  |  |
| Stage I (pre-adolescent) | 13 | 0.64 | 20.3 (11.8-34.9) |
| Stage II to IV (adolescent) | 34 | 1.35 | 25.1 (17.9-35.1) |
| Stage V (mature) | 16 | 0.37 | 43.6 (26.7-71.2) |
| Known TB contact, household or otherwise |  |  |  |
| No | 62 | 2.30 | 27.0 (21.0-34.5) |
| Yes | 1 | 0.06 | 17.3 (2.4-123.3) |

Abbreviations: QFT, interferon gamma release assay (QuantiFERON-TB®); TB, tuberculosis disease; PY, person-years; person-years calculated from date of study enrolment (staggered entry by 6 months for those with prevalent TB); censored at the last known date alive prior to 31^st^ October 2020

- 2 participants had missing data

**SUPPLEMENTAL TABLE 4. Sensitivity analysis I: variation in incidence of tuberculosis disease (TB) overall and by HIV status, using varying definitions of TB events and duration of person-time**

|  | **Total (N=599)** | **HIV+ youth (N=496)** | **HIV- youth (N=103)** | **Absolute difference in IR (95% CI), HIV+ vs HIV-** | **IRR (95% CI) comparing HIV+ to HIV-** |
| --- | --- | --- | --- | --- | --- |
| **Incidence A** |  |  |  |  |  |
| Events (all TB diagnoses, including recurrent events) | 35 | 34 | 1 | - | - |
| Person-years (censored at last known time alive)^1^ | 1840.6 | 1512.3 | 328.2 | - | - |
| Incidence of TB per 100 person-years (PY) | 1.9/100PY | 2.2/100PY | 0.3/100PY | 1.9 (1.0-2.9)/100PY | 7.38 (1.24-299.9) |
| **Incidence B** |  |  |  |  |  |
| Events (only confirmed TB diagnoses, including recurrent events) | 20 | 19 | 1 | - | - |
| Person-years (censored at last known time alive)^1^ | 1840.6 | 1512.3 | 328.2 | - | - |
| Incidence of TB per 100 person-years (PY) | 1.1/100PY | 1.3/100PY | 0.3/100PY | 0.9 (0.1-1.8)/100PY | 4.12 (0.65-171.3) |
| **Incidence C** |  |  |  |  |  |
| Events (all TB diagnoses, including recurrent events) | 35 | 34 | 1 | - | - |
| Person-years at risk (censored at last known time alive, with 6 months subtracted per incident TB event)^1^ | 1823.1 | 1495.3 | 327.7 | - | - |
| Incidence of TB per 100 person-years (PY) | 1.9/100PY | 2.3/100PY | 0.3/100PY | 2.0 (1.0-2.9)/100PY | 7.45 (1.25-302.9) |
| **Incidence D** |  |  |  |  |  |
| Events (only confirmed TB diagnoses, including recurrent events) | 20 | 19 | 1 | - | - |
| Person-years at risk (censored at last known time alive, 6 months subtracted for each incident TB event)^1^ | 1830.6 | 1502.8 | 327.7 | - | - |
| Incidence of TB per 100 person-years (PY) | 1.1/100PY | 1.3/100PY | 0.3/100PY | 1.0 (0.1-1.8)/100PY | 4.14 (0.66-172.2) |

^1^For all person-time, entry into risk-set is staggered by 6 months for those who presented with prevalent TB (ie with TB diagnosis and on TB treatment at enrolment visit)

**SUPPLEMENTAL TABLE 5. Sensitivity analysis II: TB occurrence and predictors among APHIV: all TB diagnoses versus bacteriologically confirmed TB diagnoses only, using negative binomial regression with GEE to estimate overall TB incidence, including recurrent events [incidence rate ratios]**

| **Characteristic** | **All TB diagnoses (confirmed and probable)** | | **Confirmed TB disease only (positive culture)** | |
| --- | --- | --- | --- | --- |
|  | **Incidence rate/100PY (95% CI)** | **IRR (95% CI)** | **Incidence rate/100PY (95% CI)** | **IRR (95% CI)** |
| HIV-positive (all) | 2.2 (1.6-3.1) | **-** | 1.3 (0.80-1.97) | - |
| HIV viral load log_10_ copies/mL (per log_10_ increase) | - | **1.53 (1.14-2.05)** | - | 1.38 (0.93-2.05) |
| HIV viral suppression categories |  |  |  |  |
| HIV-viral load ≤ 40 copies/mL (reference) | 1.6 (1.0-2.6) | 1.00 | 1.0 (0.5-1.8) | 1.00 |
| HIV-viral load >40, <1000 copies/mL | 2.8 (1.4-5.6) | 1.71 (0.77-3.79) | 1.8 (0.7-4.2) | 1.83 (0.67-7.04) |
| HIV-viral load ≥ 1000 copies/mL | 4.7 (2.5-9.1) | **2.90 (1.28-6.60)** | 2.1 (0.8-5.6) | 2.18 (0.68-7.04) |
| CD4 cell count categories |  |  |  |  |
| CD4 <500 cells/µL (reference) | 1.8 (1.1-2.7) | 1.00 | 1.3 (0.8-2.1) | 1.00 |
| CD4 ≥500 cells/µL | 4.1 (2.4-7.0) | **2.32 (1.17-4.60)** | 1.2 (0.5-3.3) | 1.00 (0.37-2.73) |
| Age at ART initiation |  |  |  |  |
| ≤ 2 years (reference) | 0.5 (0.1-2.1) | 1.00 | 0.3 (0.03-1.9) | 1.00 |
| >2, <6 years | 2.0 (1.1-3.5) | 3.80 (0.85-17.02) | 0.7 (0.2-1.8) | 2.52 (0.26-24.92) |
| ≥6 years | 3.5 (2.2-5.6) | **6.67 (1.54-28.97)** | 2.5 (1.5-4.4) | **9.30 (1.19-72.84)** |
| Age at study visit (per year increase) | - | **1.37 (1.15-1.67)** | - | **1.37 (1.05-1.78)** |
| Tanner staging at study visit (time-varying) |  |  |  |  |
| Stage I (pre-adolescent), reference | 1.3 (0.5-3.2) | 1.00 | 0.5 (0.1-2.1) | 1.00 |
| Stage II to IV (adolescent) | 2.4 (1.5-3.6) | 1.79 (0.73-4.36) | 1.4 (0.8-2.4) | 2.52 (0.62-10.17) |
| Stage V (mature) | 3.3 (1.6-6.6) | 2.49 (0.72-8.67) | 2.1 (0.9-4.9) | 3.85 (0.60-25.54) |
| QFT test result at enrolment |  |  |  |  |
| QFT negative (reference) | 2.6 (1.7-3.8) | 1.00 | 1.3 (0.8-2.3) | 1.00 |
| QFT positive | 1.3 (0.6-2.8) | 0.49 (0.20-1.21) | 0.9 (0.3-2.3) | 0.62 (0.19-1.97) |
| QFT results not available | 3.8 (1.2-11.9) | 1.49 (0.34-6.49) | 2.6 (0.6-10.3) | 1.91 (0.25-14.42) |

Abbreviations: Cox PH, proportional hazards regression model; TB, tuberculosis disease; PY, person-years; IRR, incidence rate ratio; CI, confidence interval; QFT, interferon gamma release assay (QuantiFERON-TB®); CD, cluster of differentiation; mL, milliliter; uL, microliter; ART, triple agent antiretroviral therapy

**SUPPLEMENTAL TABLE 6. Sensitivity analysis III: Predictors of time to first TB event among HIV+ youth: all TB diagnoses versus bacteriologically confirmed TB diagnoses only, using Cox proportional hazards regression**

|  | **All TB diagnoses (confirmed and unconfirmed)** | **Bacteriologically confirmed TB diagnoses only** |
| --- | --- | --- |
|  | **Crude HR (95% CI)** | **Crude HR (95% CI)** |
| HIV viral load log_10_ copies/mL (per log_10_ increase) | **1.41 (1.00-1.98)** | 1.25 (0.73-2.12) |
| HIV viral suppression categories |  |  |
| HIV+, viral load ≤ 40 copies/mL | 1.00 | 1.00 |
| HIV+, viral load >40, <1000 copies/mL | 1.41 (0.59-3.40) | 1.46 (0.46-4.61) |
| HIV+, viral load ≥ 1000 copies/mL | 1.96 (0.76-5.04) | 1.35 (0.29-6.29) |
| CD4 cell count categories |  |  |
| CD4 ≥500 cells/µL | 1.00 | 1.00 |
| CD4 <500 cells/µL | **2.35 (1.10-5.03)** | 1.00 (0.28-3.60) |
| Age at study visit (per year increase) | **1.33 (1.06-1.68)** | **1.43 (1.03-2.01)** |
| QFT test result at enrolment |  |  |
| QFT negative | 1.00 | 1.00 |
| QFT positive | 0.64 (0.26-1.60) | 0.92 (0.28-2.98) |
| QFT results not available | 1.66 (0.38-7.22) | 1.42 (0.18-11.26) |
| Age at ART initiation |  |  |
| ≤ 2 years | 1.00 | 1.00 |
| >2, <6 years | 3.50 (0.78-15.79) | 1.89 (0.20-18.16) |
| ≥6 years | **5.68 (1.30-24.87)** | 7.60 (0.97-59.27) |

Abbreviations: Cox PH, proportional hazards regression model; TB, tuberculosis disease; PY, person-years; HR, hazard ratio; IRR, incidence rate ratio; CI, confidence interval; HR, hazard ratio; IRR, incidence rate ratio; QFT, interferon gamma release assay (QuantiFERON-TB®); CD, cluster of differentiation; mL, milliliter; uL, microliter; ART, triple agent antiretroviral therapy

**SUPPLEMENTAL TABLE 7a. Incidence rates of all tuberculosis disease events over follow-up, comparing APHIV to HIV+ youth: crude and adjusted incidence rate ratios from negative binomial regression with generalized estimating equations**

|  | **TB Events** | **Person-years** | **Rate/100PY (95% CI)** | **Crude IRR (95% CI)** | **Adjusted IRR (95% CI)^1^** |
| --- | --- | --- | --- | --- | --- |
| Full cohort (both HIV+ and HIV- youth) | 35 | 1840.6 | 1.9 (1.4-2.6) | - | - |
| HIV status |  |  |  |  |  |
| HIV-negative (reference) | 1 | 328.2 | 0.3 (0.04-2.2) | 1.00 | - |
| HIV-positive | 34 | 1512.3 | 2.2 (1.6-3.1) | **7.36 (1.01-53.55)** | - |
| HIV status and/or HIV viral suppression categories |  |  |  |  |  |
| HIV-negative (reference) | 1 | 328.2 | 0.3 (0.04-2.2) | 1.00 | 1.00 |
| HIV-positive, viral load ≤ 40 copies/mL | 17 | 1036.8 | 1.6 (1.0-2.6) | 5.39 (0.72-40.02) | 4.92 (0.60-40.22) |
| HIV-positive, viral load >40, <1000 copies/mL | 8 | 285.5 | 2.8 (1.4-5.6) | **9.23 (1.17-72.72)** | 7.94 (0.93-67.78) |
| HIV-positive, viral load ≥ 1000 copies/mL | 9 | 190.1 | 4.7 (2.5-9.1) | **15.64 (1.94-125.92)** | **12.99 (1.46-115.68)** |
| HIV status and/or CD4 cell count categories |  |  |  |  |  |
| HIV-negative (reference) | 1 | 328.2 | 0.3 (0.04-2.2) | 1.00 | - |
| HIV-positive, CD4 ≥500 cells/μL | 21 | 1192.3 | 1.8 (1.1-2.7) | 5.76 (0.78-42.67) | - |
| HIV-positive, CD4 <500 cells/μL | 13 | 320.0 | 4.1 (2.4-7.0) | **13.36 (1.76-101.5)** | - |
| Sex |  |  |  |  |  |
| Female (reference) | 19 | 925.0 | 2.0 (1.3-3.2) | 1.00 | - |
| Male | 16 | 915.6 | 1.7 (1.1-2.9) | 0.85 (0.41-1.78) | - |
| Age at study visit (time-varying, per year increase) | - | - | - | **1.40 (1.17-1.68)** | **1.37 (1.12-1.68)** |
| Categories of age at study visit (time-varying, in years) |  |  |  |  |  |
| <12 years (reference) | 2 | 338.3 | 0.6 (0.1-2.4) | 1.00 | - |
| ≥ 12, <14 years | 10 | 646.6 | 1.5 (0.8-2.9) | 2.62 (0.58-11.9) | - |
| ≥ 14, <16 years | 14 | 604.4 | 2.3 (1.4-3.9) | 3.92 (0.90-17.14) | - |
| ≥ 16 years | 9 | 251.2 | 3.6 (1.9-6.9) | **6.06 (1.22-30.15)** | - |
| BMI categories (time-varying) |  |  |  |  |  |
| Normal weight, ≥ 18, <25 kg/m^2^ (reference) | 15 | 803.3 | 1.9 (1.1-3.1) | 1.00 | - |
| Underweight, <18 kg/m^2^ | 18 | 867.1 | 2.1 (1.3-3.3) | 1.11 (0.54-2.30) |  |
| Overweight/obese, ≥ 25 kg/m^2^ | 2 | 170.1 | 1.2 (0.3-4.7) | 0.63 (0.16-2.42) | - |
| Tanner staging at study visit (time-varying) |  |  |  |  |  |
| Stage I (pre-adolescent), reference | 5 | 445.3 | 1.1 (0.5-2.7) | 1.00 | 1.00 |
| Stage II to IV (adolescent) | 22 | 1090.1 | 2.0 (1.3-3.1) | 1.78 (0.73-4.34) | 0.92 (0.31-2.72) |
| Stage V (mature) | 8 | 305.2 | 2.6 (1.3-5.2) | 2.32 (0.66-8.10) | 0.90 (0.24-3.31) |
| Known TB contact, household or otherwise |  |  |  |  |  |
| No (reference) | 34 | 1798.8 | 1.9 (1.4-2.6) | 1.00 | - |
| Yes | 1 | 41.8 | 2.4 (0.3-17.0) | 1.26 (0.18-8.82) | - |
| QFT result at study enrolment |  |  |  |  |  |
| QFT-negative (reference) | 26 | 1176.9 | 2.2 (1.5-3.2) | 1.00 | - |
| QFT-positive | 6 | 548.7 | 1.1 (0.5-2.4) | 0.49 (0.20-1.20) | - |
| QFT test results unknown | 3 | 115.0 | 2.6 (0.8-8.1) | 1.18 (0.27-5.23) | - |

Abbreviations: TB, tuberculosis disease; PY, person-years; IRR, incidence rate ratio from population-averaged negative binomial regression with generalized estimating equations and robust variance; QFT - interferon gamma release assay (QuantiFERON-TB);  ^1^multivariable model adjusted for all variables with adjusted estimates shown as well as baseline measures of relative poverty

**SUPPLEMENTAL TABLE 7b. Incidence of all tuberculosis disease events among APHIV, stratified by participant characteristics: crude and adjusted incidence rate ratios from negative binomial regression with generalized estimating equations**

|  | **TB Events** | **Person-years** | **Rate/100PY (95% CI)** | **Crude IRR (95% CI)** | **Adjusted IRR (95% CI)^1^** |
| --- | --- | --- | --- | --- | --- |
| All HIV+ youth | 34 | 1512.3 | 2.2 (1.6-3.1) | **-** | - |
| HIV viral load (log_10_ copies/mL) | - | - | - | **1.53 (1.14-2.05)** |  |
| HIV viral suppression categories (time-varying) |  |  |  |  |  |
| HIV-viral load ≤ 40 copies/mL (reference) | 17 | 1036.8 | 1.6 (1.0-2.6) | 1.00 | 1.00 |
| HIV-viral load >40, <1000 copies/mL | 8 | 285.5 | 2.8 (1.4-5.6) | 1.71 (0.77-3.79) | 1.58 (0.71-3.52) |
| HIV-viral load ≥ 1000 copies/mL | 9 | 190.1 | 4.7 (2.5-9.1) | **2.90 (1.28-6.60)** | **2.45 (1.08-5.55)** |
| CD4 cell count (per 100 cells/uL) | - | - | - | 0.97 (0.87-1.07) | 0.97 (0.86-1.10) |
| CD4 cell count categories |  |  |  |  |  |
| CD4 ≥ 500 cells/uL (reference) | 21 | 1192.3 | 1.8 (1.1-2.7) | 1.00 | - |
| CD4 <500 cells/uL | 13 | 320.0 | 4.1 (2.4-7.0) | **2.32 (1.17-4.60)** | - |
| Age at ART initiation |  |  |  |  |  |
| ≤ 2 years (references) | 2 | 377.4 | 0.5 (0.1-2.1) | 1.00 | - |
| >2, <6 years | 12 | 597.0 | 2.0 (1.1-3.5) | 3.80 (0.85-17.02) | - |
| ≥6 years | 18 | 512.2 | 3.5 (2.2-5.6) | **6.67 (1.54-28.97)** | - |
| Sex |  |  |  |  |  |
| Female (reference) | 18 | 741.6 | 2.4 (1.5-3.8) | 1.00 | - |
| Male | 16 | 770.7 | 2.1 (1.3-3.4) | 0.85 (0.41-1.80) | - |
| Age at study visit (time-varying, per year increase) | - | - | - | **1.37 (1.15-1.67)** | **1.32 (1.06-1.65)** |
| Categories of age at study visit (time-varying, in years) |  |  |  |  |  |
| <12 years (reference) | 2 | 262.2 | 0.8 (0.2-3.0) | 1.00 | - |
| ≥ 12, <14 years | 10 | 543.7 | 1.8 (1.0-3.4) | 2.42 (0.53-11.00) | - |
| ≥ 14, <16 years | 14 | 501.9 | 2.8 (1.6-4.7) | 3.67 (0.84-16.03) | - |
| ≥ 16 years | 8 | 204.4 | 3.9 (2.0 -7.8) | 5.15 (1.00-26.47) | - |
| BMI categories (time-varying) |  |  |  |  |  |
| Normal weight, ≥ 18, <25 kg/m^2^  (reference) | 14 | 650.6 | 2.2 (1.3-3.6) | 1.00 | - |
| Underweight, <18 kg/m^2^ | 18 | 747.9 | 2.4 (1.5-3.8) | 1.12 (0.53-2.34) | - |
| Overweight/obese, ≥ 25 kg/m^2^ | 2 | 113.7 | 1.8 (0.4-7.0) | 0.82 (0.21-3.14) | - |
| Tanner staging at study visit (time-varying) |  |  |  |  |  |
| Stage I (pre-adolescent), reference | 5 | 380.5 | 1.3 (0.5-3.2) | 1.00 | 1.00 |
| Stage II to IV (adolescent) | 21 | 888.7 | 2.4 (1.5-3.6) | 1.79 (0.73-4.36) | 0.93 (0.31-2.78) |
| Stage V (mature) | 8 | 243.1 | 3.3 (1.6-6.6) | 2.49 (0.72-8.67) | 1.00 (0.27-3.64) |
| Known TB contact, household or otherwise |  |  |  |  |  |
| No (reference) | 33 | 1485.1 | 2.2 (1.6-3.1) | 1.00 | - |
| Yes | 1 | 27.2 | 3.7 (0.5-26.1) | 1.65 (0.25-11.00) | - |
| QFT result at study enrolment |  |  |  |  |  |
| QFT-negative (reference) | 25 | 967.0 | 2.6 (1.7-3.8) | 1.00 | - |
| QFT-positive | 6 | 467.4 | 1.3 (0.6-2.8) | 0.49 (0.20-1.21) | - |
| QFT test results unknown | 3 | 77.9 | 3.8 (1.2-11.9) | 1.49 (0.34-6.49) | - |

Abbreviations: TB, tuberculosis disease; PY, person-years; IRR, incidence rate ratio from population-averaged negative binomial regression with generalized estimating equations and robust variance; QFT - interferon gamma release assay (QuantiFERON-TB); ^1^multivariable model adjusted for all variables with adjusted estimates shown as well as baseline measures of relative poverty
